# Dynamics and energy encoding of a star-like neuron network composed of the Wang-Zhang model induced by compressing a sphere into a fingertip

**DOI:** 10.1007/s11571-026-10436-0

**Published:** 2026-03-19

**Authors:** Xuerong Shi, Wanjiang Xu, Lizhou Zhuang, Fanqi Meng, Haibo Jiang, Zuolei Wang

**Affiliations:** School of Mathematics and Statistics, Yancheng Teachers University, Yancheng, 224002 China

**Keywords:** Re Synchronization, Energy encoding, Star-like neuron network, Wang-Zhang model

## Abstract

The information transmission of the tactile system is closely related to neuron network dynamics and energy metabolism. However, the correlation mechanism between neuron encoding and energy consumption for fingertip under compression remains unclear. In this study, a star-like neuron network is constructed using the Wang-Zhang model as the node, and it is combined with a contact mechanics model to simulate the phenomenon when a sphere being compressed into the fingertip. The remote synchronization characteristics is explored via average maximum correlation coefficient and Kuramoto order parameter, and energy encoding rules of the network are discussed. The results show that the star-like network can achieve remote synchronization between central and peripheral neurons. The energy consumption of central neurons is much higher than that of peripheral neurons due to signal integration and direct compression. The neuron energy consumption exhibits a spatial distribution of “high in the center and low in the periphery”. It is found that there is an optimal value for the number of network layers, at which energy consumption and information processing efficiency reach a balance. This study reveals the neurometabolic mechanism of tactile perception and provides a new theoretical reference for the study of tactile neuronal encoding.

## Introduction

Sensation is the “nutrition” of the nervous system. Without various sensory stimuli and information input, the nervous system cannot respond to the outside world, and therefore cannot develop various complex functions appropriately. The important medium for generating sensation is the tactile system composed of countless tactile receptors and helps us transmit information about the materials, shapes, volumes, sizes, temperatures, and other properties of various objects in the surrounding environment to the brain. Results (Phillips and Johnson 1981a; Johnson 2001) indicated that the sensitivity of living organisms to external stimuli is dependent on the functionality of skin microstructure composing of many tactile receptors. These mechanoreceptors exhibited sensitivity to the contacted object(Johansson et al. 1982). Therefore, exploring the behavior properties of tactile receptors under external stimuli is an interesting topic. It is the origin of our work.

To investigate the cluster dynamics of neurons, various neuron networks were proposed. A fully connected neural network is established and the quantitative relationship among synchronous oscillation, energy consumption, and network parameters was explained(Zhu et al. 2018). A small-world network is considered by coupling Izhikevich models and the collective behaviors of the network were studied (Qu and Wang 2017). A small-world network of Hodgkin-Huxley neurons was constructed and the effect of shortcut probability on the spike of the network was discussed (Batista et al.^2014^). A double-layer heterogeneous network involving two interacting sub-networks was addressed and the fast-slow-mode Hopf bifurcation was analyzed(Liu et al. 2024). A fraction-order heterogeneous network was presented by coupling FN neurons with two HR neurons and the factors influencing the network dynamics were explored(Ding et al. 2024). A ring network was constructed by coupling heterogeneous tonic and bursting neurons via the Huber-Braun model, and the effect of neuronal heterogeneity on firing pattern transitions in the network was investigated(Agustin et al 2024). A small-world network based on rodent dense neocortical microcircuit (NMC) was developed to explain neurodegenerative diseases linked to region-specific neuron type loss (Gal et al. 2021). In (Joseph et al. 2024), the regular network, small-world network, and random network composed of Chay neurons were studied, and then coherent, incoherent, and imperfect chimera patterns were found in the networks. These studies mainly focus on the dynamics of neuron networks, failing to reveal the intrinsic mechanisms of the complex dynamics of neural networks. Meanwhile, the collective dynamics of biological nervous systems (such as synchronization and spatiotemporal coding) are closely related to cellular energy metabolism, such as ion gradient maintenance and synaptic transmission, which rely on ATP for energy supply, but existing research has not paid enough attention to the energy changes accompanying the dynamical behaviors of neural networks, making it difficult to fully explain the information processing logic of the nervous system.

Actually, the dynamics of a neuron network is a process of information processing and signal transmission. To study the mechanism of information processing of neurons, various neuron information coding have been developed (Nicholas and Garrett 2004; Wang et al. 2009; Moujahid et al.^2011^; Zhang et al. 2014; Intveld et al. 2018; Wong 2020; Elliott 2022; Li et al.^2024^). As one of the challenging research topics (Shunichi and Hiroyuki 2005), neural coding and decoding are important tools for neuroscience research. Research results(Levy and Baxter 1996, 2002) indicated that neural coding correlates strongly with energy consumption. Synchronization along with the accompanying energy changing in the neuron network was discovered (An et al. 2024). Research into the cortical network revealed energy distribution strongly correlates with neural network synchronous oscillation(Wang and Wang 2014). It is pointed out that biophysical function activation and biological neuron adaptation may rely on energy flow (Ma 2023). In (Yang and Ma 2023), the authors discussed the adaptive synchronization in chain and ring neuron networks as well as the corresponding energy diversity and found that energy injection and regulation effectively control synchronous patterns in neuron networks. These studies addressed that neuron synchronization is accompanied by energy changes, and energy distribution in cortical networks is closely related to synchronous oscillations. However, the specific relationship between neuron coding and energy consumption in tactile stimulation scenarios (such as fingertip pressure) remains unclear. When the fingertip is compressed by an object, subcutaneous neurons exhibit diverse firing patterns and trigger energy changes, but the relationship between these firing patterns, network synchronization, and energy metabolism still lacks in-depth exploration.

In the previous results, it was found that the tactile system transmits environmental information via mechanoreceptors, various neural networks (fully connected, small-world) are provided with cluster dynamics, and neural coding strongly correlates with energy consumption. The specific neuron coding-energy consumption relationship in tactile stimulation (e.g., fingertip pressure) remains unclear. Quasar networks, due to their central integration characteristics, align with the principles of efficient information processing and energy optimization in biological nervous systems.

Activated by the above, a star-like neuron network is to be constructed. The remote synchronization and energy encoding in a star-like network under fingertip compression with spherical object will be focused on, which is critical for uncovering the neuro-metabolic mechanisms of tactile perception. Other parts of this paper are arranged as follows. The contact mechanics model and Wang-Zhang model are introduced in Sect. "Contact mechanics model", Sect. "Wang-Zhang neuron model", respectively. In Sect. "Star-like neuron network", Star-like neuron network is constructed. Simulation results are depicted in Sect. "Simulations". Conclusions are drawn in Sect. "Results".

## Contact mechanics model

According to (Sripati et al. 2006), the contact pressure at the interface of two objects is given by Eq. (1), and vertical displacement of the body surface is described by Eq. (2).

1 $$ p\left( {r,d} \right) = \frac{{2E^{*} }}{{R^{*} }}\left( {a_{H}^{2} - r^{2} } \right)^{{{\raise0.7ex\hbox{$1$} \!\mathord{\left/ {\vphantom {1 2}}\right.\kern-0pt} \!\lower0.7ex\hbox{$2$}}}} e^{{{\raise0.7ex\hbox{${ - r^{2} F}$} \!\mathord{\left/ {\vphantom {{ - r^{2} F} {2.67a_{H} }}}\right.\kern-0pt} \!\lower0.7ex\hbox{${2.67a_{H} }$}}}} $$

2 $$U\left(r\right)=\left\{\begin{array}{c}\pi {p}_{H}\frac{2{a}_{H}^{2}-{r}^{2}}{2{a}_{H}{E}^{*}},0\le r<{a}_{H}\\ \frac{{p}_{H}}{{2a}_{H}{E}^{*}}\left[\left(2{a}_{H}^{2}-{r}^{2}\right){sin}^{-1}\left({a}_{H}/r\right)+\frac{r{a}_{H}}{2}\sqrt{1-{\left({a}_{H}/r\right)}^{2}}\right],r\ge {a}_{H}\end{array}\right.$$

where $${R}^{*}$$ is the equivalent radius satisfying $${\raise0.7ex\hbox{$1$} \!\mathord{\left/ {\vphantom {1 {R^{*} }}}\right.\kern-0pt} \!\lower0.7ex\hbox{${R^{*} }$}} = {\raise0.7ex\hbox{$1$} \!\mathord{\left/ {\vphantom {1 {R_{skin} }}}\right.\kern-0pt} \!\lower0.7ex\hbox{${R_{skin} }$}} + {\raise0.7ex\hbox{$1$} \!\mathord{\left/ {\vphantom {1 {R_{mat} }}}\right.\kern-0pt} \!\lower0.7ex\hbox{${R_{mat} }$}}$$ , $${a}_{H}=\sqrt{{R}^{*}d}$$ is the contact radius, $$F=4{E}^{*}\sqrt{{R}^{*}{d}^{3}}/3$$ denotes the force on the sphere, $${p}_{H}=3F/2\pi {R}^{*}d$$ represents the pressure at the contact center, $$r$$ represents the distance to the force point, $$U(r)$$ denotes the skin shape variable at $$r$$ mm from the force application point, and $${E}^{*}$$ is the equivalent Young’s modulus of two contact objects, which can be calculated via formula.

3 $$ 1/E^{*} = {\raise0.7ex\hbox{${\left( {1 - v_{skin}^{2} } \right)}$} \!\mathord{\left/ {\vphantom {{\left( {1 - v_{skin}^{2} } \right)} {E_{skin} }}}\right.\kern-0pt} \!\lower0.7ex\hbox{${E_{skin} }$}} + {\raise0.7ex\hbox{${\left( {1 - v_{mat}^{2} } \right)}$} \!\mathord{\left/ {\vphantom {{\left( {1 - v_{mat}^{2} } \right)} {E_{mat} }}}\right.\kern-0pt} \!\lower0.7ex\hbox{${E_{mat} }$}} $$

Research in (Kim et al. 2010) suggested that strain energy density of MCNC (Merkel cell-neurite complex) under pressure is tightly linked to firing rate of SAI (slowly adapting type I) mechanoreceptor. When the skin is compressed, the neuron of skin can be stimulated by external stimuli induced by deformation. Existing results (Kim et al. 2010; Gerling and Thomas 2008) revealed the external stimulus properties using energy transformation.

4 $${U}_{0}(r)=\frac{3}{4G}{\tau }_{oct}^{2}(r)$$

where $r$ denotes distance from the force application point, ${U}_{0}(r)$ represents the strain energy density at the point. ${\tau }_{oct}(r)$ means the octahedral shear stress and can be calculated by.

5 $${\tau }_{oct}=\frac{1}{3}\sqrt{{({\sigma }_{xx}-{\sigma }_{yy})}^{2}+{({\sigma }_{yy}-{\sigma }_{zz})}^{2}+{({\sigma }_{xx}-{\sigma }_{zz})}^{2}+{6(\sigma }_{xy}^{2}+{\sigma }_{yz}^{2}+{\sigma }_{xz}^{2}})$$

where ${\sigma }_{xx}$, ${\sigma }_{yy}$, and ${\sigma }_{zz}$ are normal stresses, ${\sigma }_{xy}$, ${\sigma }_{yz}$, ${\sigma }_{xz}$ are shear stresses. $G$ is shear modulus of elasticity given by

6 $$G=\frac{E}{1+v}$$

with $E$, $v$ being the skin’s Young’s modulus and the Poisson’s ratio of the skin, respectively.

Additionally, ${\sigma }_{xx}$, ${\sigma }_{yy}$, ${\sigma }_{zz}$ and ${\sigma }_{xy}$, ${\sigma }_{yz}$, ${\sigma }_{xz}$ can be calculated by.

7 $$ \begin{aligned} & \left( {\begin{array}{*{20}c} {\sigma _{{xx}} } & {\sigma _{{xy}} } & {\sigma _{{xz}} } \\ {\sigma _{{xy}} } & {\sigma _{{yy}} } & {\sigma _{{yz}} } \\ {\sigma _{{xz}} } & {\sigma _{{yz}} } & {\sigma _{{zz}} } \\ \end{array} } \right) = \frac{{p\left( r \right)}}{{2\pi }}\left( {\begin{array}{*{20}c} {{\mathrm{cos}}\theta } & { - {\mathrm{sin}}\theta } & 0 \\ {{\mathrm{sin}}\theta } & {{\mathrm{cos}}\theta } & 0 \\ 0 & 0 & 1 \\ \end{array} } \right) \\ & \left( {\begin{array}{*{20}c} {\frac{{3r^{2} z}}{{R^{5} }} - \frac{{1 - v}}{{R^{2} + zR}}} & 0 & {\frac{{3rz^{2} }}{{R^{5} }}} \\ 0 & {(1 - 2v)(\frac{1}{{r^{2} }} - \frac{z}{{r^{2} R}} - \frac{z}{{R^{3} }})} & 0 \\ {\frac{{3rz^{2} }}{{R^{5} }}} & 0 & {\frac{{3r^{3} }}{{R^{5} }}} \\ \end{array} } \right)\left( {\begin{array}{*{20}c} {{\mathrm{cos}}\theta } & {{\mathrm{sin}}\theta } & 0 \\ { - {\mathrm{sin}}\theta } & {{\mathrm{cos}}\theta } & 0 \\ 0 & 0 & 1 \\ \end{array} } \right) \\ \end{aligned} $$

where $$R=\sqrt{{r}^{2}+{z}^{2}}$$, $$z=U\left(r\right),$$ $$\theta =\frac{\pi }{4}$$, $$r=\sqrt{{x}^{2}+{y}^{2}}$$

The sigmoidal function was found to convert SED (strain energy density) induced by external stimulation into current (Iggo and Muir 1969) and the SED-to-current(Gerling and Thomas 2008)conversion formula is given by.

8 $$I\left(r\right)=\left\{\begin{array}{c}\begin{array}{cc}\alpha \frac{1}{1+{e}^{\gamma (\lambda -{U}_{0}(r))}}& U(r)\ge 5\mu m\end{array}\\ 0\begin{array}{cc}& \end{array}\begin{array}{cc}& U\left(r\right)<5\mu m\end{array}\end{array}\right.$$

where $\alpha$, $\gamma$ and $\lambda$ are adjustable parameters for fitting the SAI mechanoreceptors.

## Wang-Zhang neuron model

**Fig. 1:**
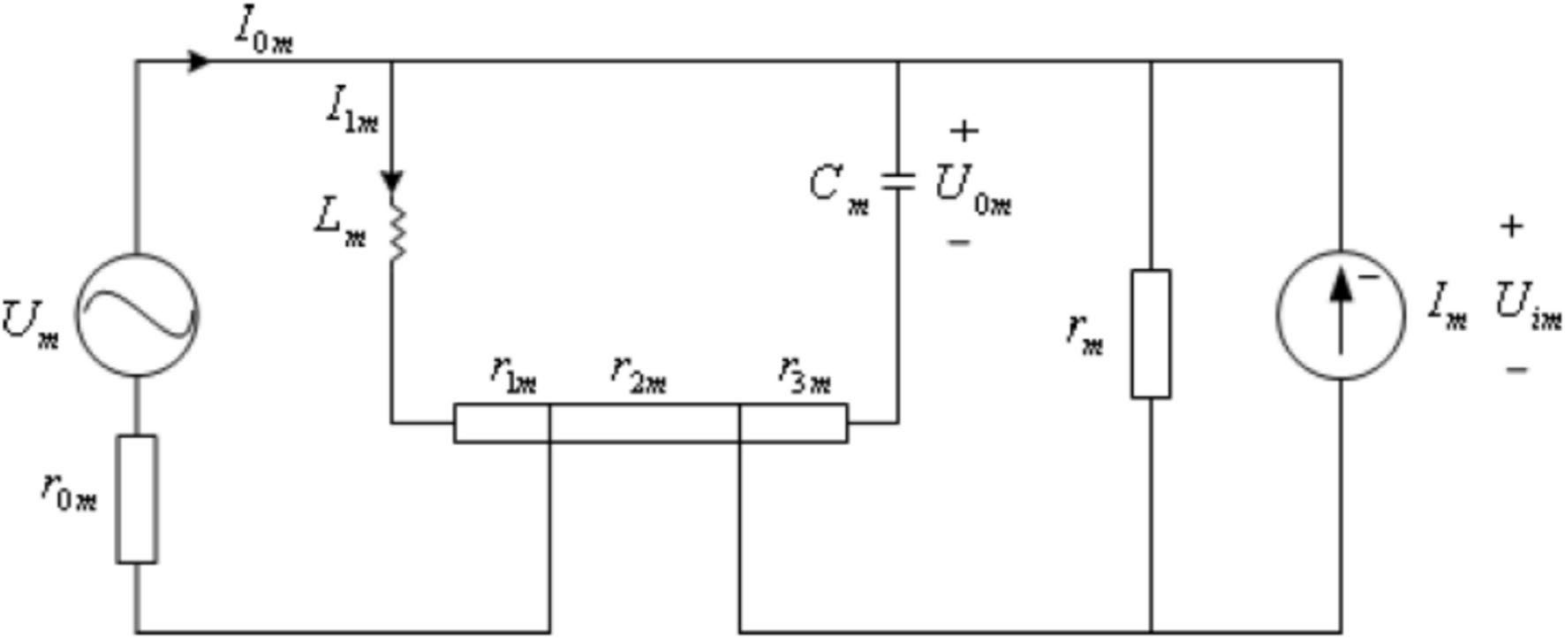
Electric energy model of Wang-Zhang neuron model

From the perspective of neuron energy calculation, a novel neuro-biophysical model was proposed by Wang-Zhang and illustrated as Fig. 1(Wang and Wang 2014), which depicts the schematic diagram of the $m{\mathrm{th}}$ neuron. $C_{m}$ denotes the membrane capacitance, $U_{m}$ is the voltage source.$I_{0m}$ is the corresponding input current, $L_{m}$ means the inductance. $I_{m}$ is current input from external neurons, $r_{0m}$ is the internal resistance of $U_{m}$. The current source and voltage source divide the membrane resistance into three parts: $r_{1m}$, $r_{2m}$ and $r_{3m}$.

According to Fig. 1, following equations (Wang and Wang 2014) can be obtained

9 $$ \left\{ {\begin{array}{*{20}c} {U_{m} = r_{0m} I_{0m} + r_{1m} I_{1m} + L_{m} \dot{I}_{1m} } \\ {I_{0m} = I_{1m} - I_{m} + \frac{{U_{im} }}{{r_{m} }} + C_{m} \dot{U}_{0m} } \\ {U_{im} = C_{m} r_{3m} \dot{U}_{0m} + U_{0m} \begin{array}{*{20}c} {} & {\begin{array}{*{20}c} {} & {} \\ \end{array} } \\ \end{array} } \\ \end{array} ,} \right. $$

10 $$ \begin{aligned} & L_{m} \dot{I}_{{1m}} + r_{{1m}} I_{{1m}} \\ & \; = C_{m} (r_{{2m}} + r_{{3m}} + \frac{{r_{{2m}} r_{{3m}} }}{{r_{m} }})\dot{U}_{{0m}} + (1 + \frac{{r_{{2m}} }}{{r_{m} }})U_{{0m}} - r_{{2m}} I_{m} \\ \end{aligned} $$

11 $$ \begin{aligned} I_{m} = & i_{{1m}} + \sum\limits_{{j = 1}}^{n} {[i_{{0m(j - 1)}} } \sin (\omega _{m} (j - 1)(t_{j} - t_{{j - 1}} ))] \\ & \; + i_{{0m(n)}} \sin (\omega _{m} (n)(t - t_{n} )) \\ \end{aligned} $$

Since power represents energy per unit time, expression

12 $$ P_{m} = U_{m} I_{0m} + U_{im} I_{m} $$

can denote the energy of the $m{\mathrm{th}}$ neuron. And then the total energy of a neuron group involving $N$ neurons can be gained as.

13 $$ P = \sum\limits_{m = 1}^{N} {P_{m} } = \sum\limits_{m = 1}^{N} {(U_{m} I_{0m} + U_{im} I_{m} )} \triangleq P(t,U_{0m} ,\dot{U}_{0m} ) $$

According to Eqs. (9)-(13), it can be obtained that

$$ \begin{aligned} & U_{{0m}} = - \frac{{\hat{g}_{1} }}{{\lambda _{m}^{2} }} - \frac{{\hat{g}_{2} e^{{ - a\left( {t - t_{n} } \right)}} }}{{\lambda _{m}^{2} - a^{2} }} - \frac{1}{{\lambda _{m}^{2} + \lambda \omega _{m}^{2} }} \times (\hat{g}_{3} {\mathrm{sin}}\omega _{m} \left( n \right)\left( {t - t_{n} } \right) \\ & \; + \hat{g}_{4} {\mathrm{cos}}\omega _{m} \left( n \right)\left( {t - t_{n} } \right) + \left( {U_{{0m}} \left( {t_{n} } \right) + \frac{{\hat{g}_{1} }}{{\lambda _{m}^{2} }} + \frac{{\hat{g}_{2} }}{{\lambda _{m}^{2} - a^{2} }} + \frac{{\hat{g}_{4} }}{{\lambda _{m}^{2} \lambda + \omega _{m}^{2} \left( n \right)}}} \right)e^{{ - \lambda _{m} \left( {t - t_{n} } \right)}} \\ & \;,t_{n} \langle t\langle t_{{n + 1}} ,n = {\mathrm{0,1}},2, \ldots ,t_{0} = 0) \\ \end{aligned} $$

where $$\lambda_{m} = \sqrt {\frac{{d_{4m} }}{{d_{1m} }}}$$,

$$ g(t) = \mathop {g_{1} }\limits^{ \wedge } + \mathop {g_{2} }\limits^{ \wedge } e^{{ - a(t - t_{n} )}} + \mathop {g_{3} }\limits^{ \wedge } \sin \omega_{m} (n)(t - t_{n} ) + \mathop {g_{4} }\limits^{ \wedge } \cos \omega_{m} (n)(t - t_{n} ) $$

$$ \left( {t_{n} < t < t_{n - 1} ,n = 0,1, \ldots ,t_{0} = 0} \right) $$

$$ \mathop {g_{1} }\limits^{ \wedge } = (g_{2} - g_{1} \frac{{r_{2m} }}{{r_{1m} }})i_{1m} + g_{2} \sum\limits_{j = 1}^{n} {i_{0m} (j - 1)} \sin \omega_{m} (j - 1)(t_{j} - t_{j - 1} ) $$

$$ \begin{gathered} \mathop {g_{2} }\limits^{ \wedge } = g_{1} (K + \frac{{r_{2m} }}{{r_{1m} }})i_{1m} e^{{ - at_{n} }} - \frac{{r_{2m} }}{{L_{m} }}g_{1} \frac{{i_{0m} (n)\omega_{m} (n)}}{{a^{2} + \omega_{m}^{2} (n)}} - \frac{{r_{2m} }}{{L_{m} }}g_{1} \hfill \\ \sum\limits_{j = 1}^{n} {[\frac{{i_{0m} (j - 1)}}{{a^{2} + \omega_{m}^{2} (j - 1)}}(e^{{ - a(t_{n} - t_{j} )}} (a\sin \omega_{m} (j - 1)(t_{j} - t_{j - 1} ) - \omega_{m} (j - 1)\cos \omega_{m} (j - 1)(t_{j} - t_{j - 1} ))} \hfill \\ + \omega_{m} (j - 1)e^{{ - \alpha (t_{n} - t_{ - 1j} )}} \hfill \\ \end{gathered} $$

$$ \mathop {g_{3} }\limits^{ \wedge } = i_{0m} (n)(g_{2} - \frac{{ar_{2m} g_{1} }}{{L_{m} (a^{2} + \omega_{m}^{2} (n))}}) $$

$$\mathop {g_{4} }\limits^{ \wedge } = i_{0m} (n)\omega_{m} (n)(g_{3} + \frac{{r_{2m} g_{1} }}{{L_{m} (a^{2} + \omega_{m}^{2} (n))}})$$

with

$$ \begin{aligned} & g_{1} = \frac{1}{{2d_{{1m}} }}(\frac{{2r_{{0m}} (r_{m} + r_{{1m}} + r_{{2m}} ) + r_{{1m}} (r_{m} + r_{{2m}} )}}{{r_{m} r_{{1m}} }} \\ & \; + aC_{m} (2r_{{0m}} + r_{{2m}} + r_{{3m}} + \frac{{(2r_{{0m}} + r_{{2m}} )r_{{3m}} }}{{r_{m} }})) \\ \end{aligned} $$

$$ \begin{aligned} & g_{2} = \frac{1}{{2d_{{1m}} }} \\ & \left[ {\frac{{C_{m} r_{{2m}} }}{{L_{m} }}(2r_{{0m}} + r_{{2m}} + r_{{3m}} + \frac{{(2r_{{0m}} + r_{{2m}} )r_{{3m}} }}{{r_{m} }} - \frac{{2r_{{0m}} (r_{m} + r_{{1m}} + r_{{2m}} }}{{r_{m} r_{{1m}} }} + Z_{m} } \right] \\ \end{aligned} $$

$$ g_{3} = \frac{{C_{m} }}{{d_{1m} }}(1 + \frac{{r_{3m} }}{{r_{m} }})(r_{0m} + r_{2m} ) $$

$$ Z_{m} = \frac{{r_{2m} (2r_{1m} + r_{m} + r_{2m} ) + r_{1m} r_{m} }}{{r_{m} r_{1m} }} $$

Results in (Wang et al. 2018) indicates that Wang-Zhang model is essentially equivalent to H–H model.

## Star-like neuron network

As important tools in computational neuroscience, neural network can be regarded as an abstraction of complex neural systems by treating neurons as nodes and their interactions as links. Consequently, various network representations have gained widespread popularity over the past decade (Jo et al. 2005; Deng et al. 2014; Qin et al. 2014; Yao et al.^2024^; Yuan et al.^2025^; Xu et al. 2023; Towlson et al.^2013^). Towlson et al. held the view that modules connect with others via highly linked hub nodes, which preferentially interact to form putative “rich clubs”—integrative cores in C. elegants (Towlson et al.^2013^). Based on neurite clustering combined with connectomic and functional data, Brittin et al. highlighted striking similarities between C. elegans’ organization and pyramidal neuron architecture in the mammalian cortex(Brittin et al. 2021). In addition, astrocytes in the brain have radial protuberances that form a star-like structure, interacting closely with the surrounding neurons. Furthermore, considering the principle of self-organization in developmental biology and embryonic development, neurons migrate to their target positions guided by a chemical gradient, forming local clustered connections (e.g., cortical columns), which forms a star-like structure. Given the centralized integration in biological nervous systems, combined with the principles of efficient information processing and energy optimization, star-like neuron network is an important structure in discussing the dynamics of the neuron system.

**Fig. 2:**
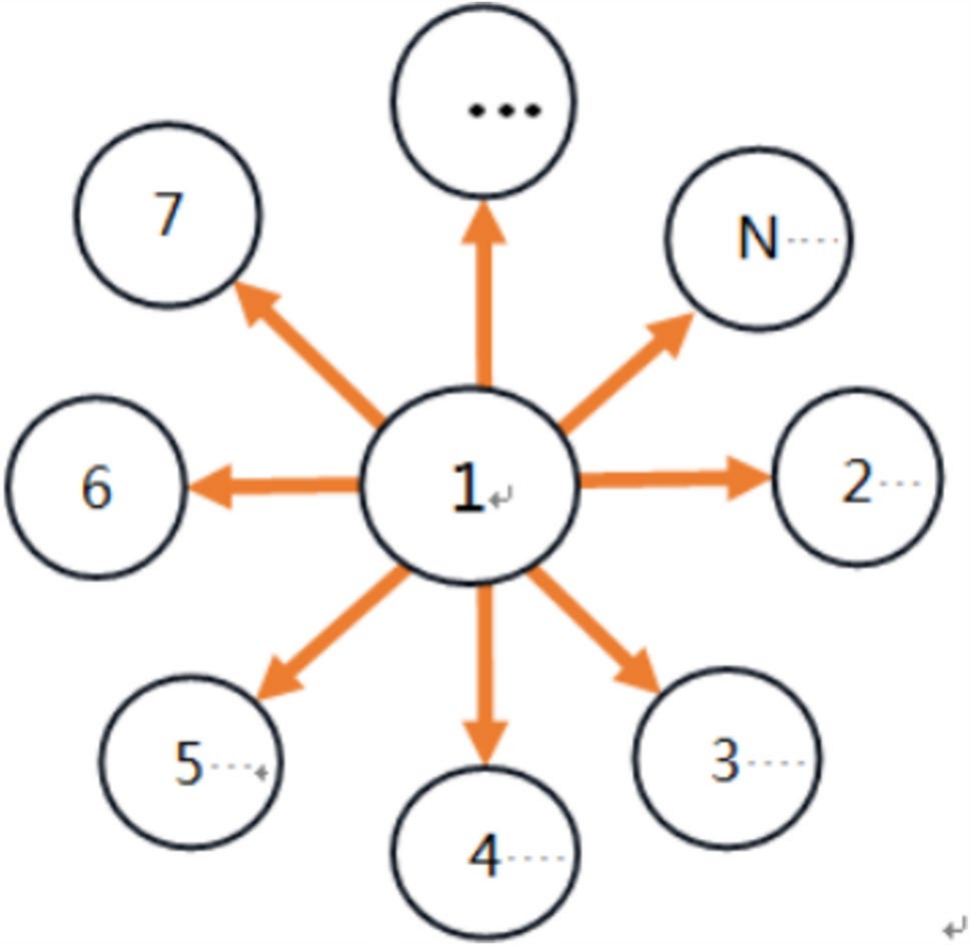
Topology of star-like network

From another aspect, the biological significance of star-like neuron network lies in its alignment with the tactile system’s information processing mode: tactile receptors in biological skin transmit signals radially with central neurons as hubs, matching the ‘‘hub-peripheral node’’ structure. This topology simulates the physiological process where neurons in the core compressed region (hub node) integrate signals and transmit them to surrounding areas (peripheral nodes), accurately reflecting energy consumption differences across deformed regions. It facilitates analyzing the hierarchical link between ‘‘deformation-signal transmission-energy consumption’’ conforming to the biological nervous system’s principles of efficient integration and energy optimization. Namely, when the neuron received certain signal, it may transit it to other neurons in a star pattern, where a neuron couples with other neurons in the form of a star model (Fig. 2). The dynamics of star-like networks mainly involves the interaction and information transmission between nodes in the network. The characteristic of this network is that there is a hub node, and other nodes are directly connected to it, but not directly connected to each other. This structure is of great significance in network science and complex system research, as it can exhibit rich dynamic behaviors, especially in the study of synchronization phenomena. ‌

Considering a star-like network with N nodes, the equations can be depicted as.

14 $$ \frac{{dV_{i} }}{dt} = f(V_{i} ) + \frac{{H_{ij} }}{{d_{i}^{{}} }}\sum\limits_{j = 1}^{N} {g_{ij} (V_{j} - V_{i} )} $$

where $V_{i}$ denotes the membrane potential of the $i{\mathrm{th}}$ node, $f(V_{i} )$ represents the dynamical function of the $i{\mathrm{th}}$ node, $d_{i}^{{}}$ is the degree of the $i{\mathrm{th}}$ node, $(g_{ij} )$ is the adjacency matrix with symmetrical features, $H_{ij}$ is the coupling strength. The adjacency matrix $(g_{ij} )$ is depicted as.

15 $$ (g_{ij} ) = \left( {\begin{array}{*{20}c} 0 & 1 & \cdots & 1 & 1 \\ 1 & 0 & \cdots & 0 & 0 \\ 1 & 0 & \cdots & 0 & 0 \\ \vdots & \vdots & \vdots & 0 & 0 \\ 1 & 0 & 0 & 0 & 0 \\ \end{array} } \right) $$

The degrees of the nodes are $[d_{i} ] = [ - (N - 1),1,1, \cdots ,1,1]$. The characteristic of each node is represented by a neuron model. The external stimulus the node neuron received involves two parts, the transduction current *I*_*tran*_ induced by displacement of skin and the stimuli current I_Σ_ transferred from other adjacent neurons, namely $I = I_{tran} + I_{{\sum {} }}$.

For the star-like neuron network, the coupling strength matrix has following characteristics, the coupling strength between the elements on the star-line are non-zero value uniformly distributed in some range, and others are zero.

## Simulations

In Eq. (14), the node dynamic function $f(V_{i} )$ incorporates core equations from the neuron model (W-Z or HH model). W-Z model substitutes Eqs. (9)–(11) (membrane potential, inductance, resistance-related equations), while the HH model uses classic sodium, potassium, and leak current equations. Network calculation steps are given as follows. (1) Obtain skin deformation-induced input current $I(r)$ via the contact mechanics model (Eqs. (1)–(8)). (2) Input $I(r)$ as external stimulation into the W-Z or HH model to generate individual node membrane potential $U_{i} (t)$. (3) Calculate inter-node coupling currents using the star-like network adjacency matrix (Eq. (15)) and coupling strength. (4) Substitute into Eq. (14) and perform numerical iteration (step size: 0.01 ms) to obtain the network’s dynamic response, then compute energy consumption using energy formulas.

To investigate energy coding in a stimulated neuron network, bidirectionally asymmetric coupling strengths are considered. Namely neuron 1 is coupled to neuron 2 with coupling strength $\omega_{12}$, and neuron 2 to neuron 1 with $\omega_{21}$($\omega_{12} \ne \omega_{21}$). we assume that asynaptic coupling strengths between neurons are statistically uniformly distributed (Rubinov et al. 2011). In the contact mechanics model, MCNC (Merkel cell-neurite complex) is considered, while the firing rates of MCNCs belonged to SA1 mechanoreceptors(Johansson et al.^1982^).Consequently, in the simulations, SA1 receptors are focused on.

**Fig. 3:**
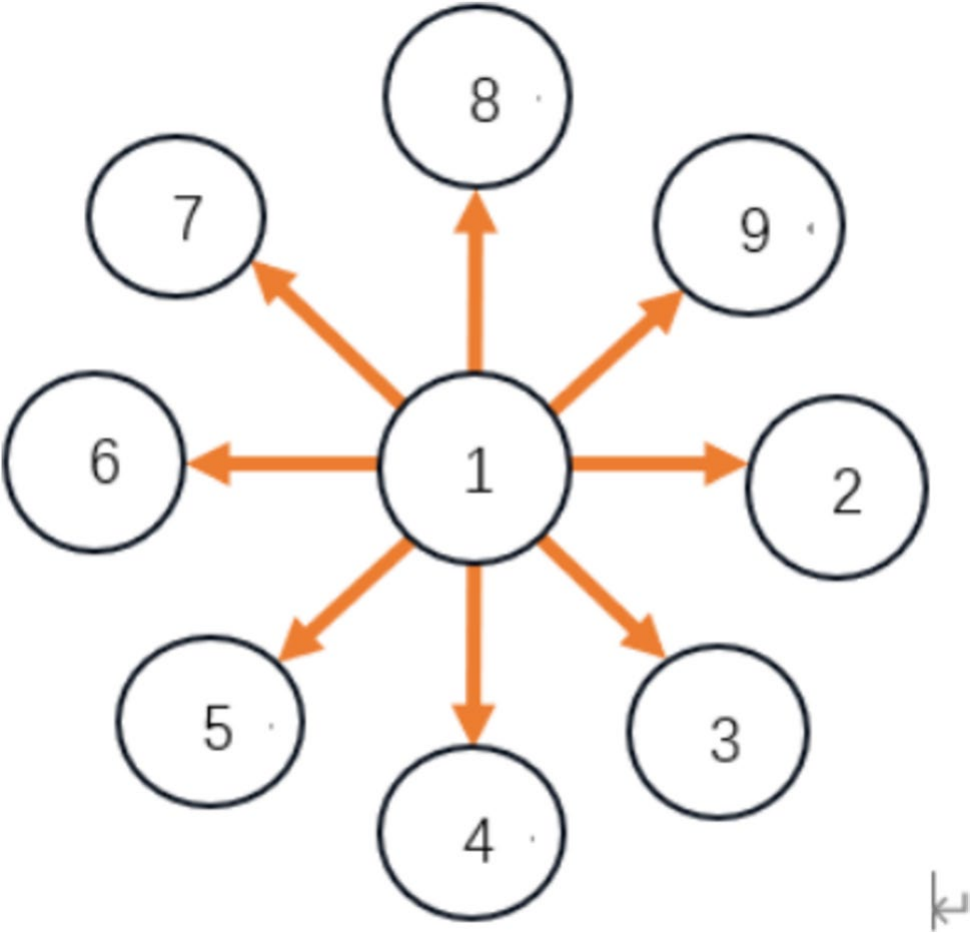
A star-like neuron network with 9 nodes

For simplicity, a star-like neuron network with 9 nodes is considered (Fig. 3). In this star-like neuron network, the first neuron is taken as the hub and other neurons are peripheral nodes. And then, the first neuron is coupled with all peripheral neurons while all peripheral neurons are not coupled with each other. Suppose $w_{ij}$ denotes the coupling between the $i{\mathrm{th}}$ neuron and the $j{\mathrm{th}}$ neuron in the star-like neuron network, the coupling matrix can be described as.

16 $$ W = \left[ {\begin{array}{*{20}c} 0 & {w_{12} } & {w_{13} } & \cdots & {w_{18} } \\ {w_{21} } & 0 & 0 & \cdots & 0 \\ {w_{31} } & 0 & 0 & \cdots & 0 \\ \vdots & \vdots & \vdots & \vdots & \vdots \\ {w_{81} } & 0 & 0 & \cdots & 0 \\ \end{array} } \right] $$

### Skin displacement vs. distance from force application point

According to the research about SAI receptor (Johansson et al.^1982^), the density of SAI input receptors under the fingertip skin is about 60–80 per square centimeter (Peters et al. 2017; Moujahid et al.^2011^). SAI input has good spatial interpretation ability for stimuli within a range of 0.5 mm. This is much smaller than SAI’s own receptive field of 2-3 mm. Therefore, SAI receptors located at different distances from the point of force application are more sensitive to external stimuli. Results in (Phillips and Johnson KO 1981a) predicted that non-zero skin displacement at all distances from the force application point theoretically, but afferent responses are unaffected by forces acting at $r_{b} > 3$ mm from the receptor.

To isolate the core effects of network topology (star-like structure) and external pressure on neural synchronization and energy encoding, the skin is assumed homogeneous, isotropic, infinite, and linearly elastic. This simplification allows us to establish a clear baseline for understanding how pressure-induced skin deformation translates to neural activity, without confounding variables from skin heterogeneity. Additionally, this assumption aligns with previous computational studies on tactile sensing (Sripati et al. 2006; Yao and Wang 2019), enabling direct comparison with existing literature and facilitating the validation of our model’s fundamental mechanisms.

**Fig. 4:**
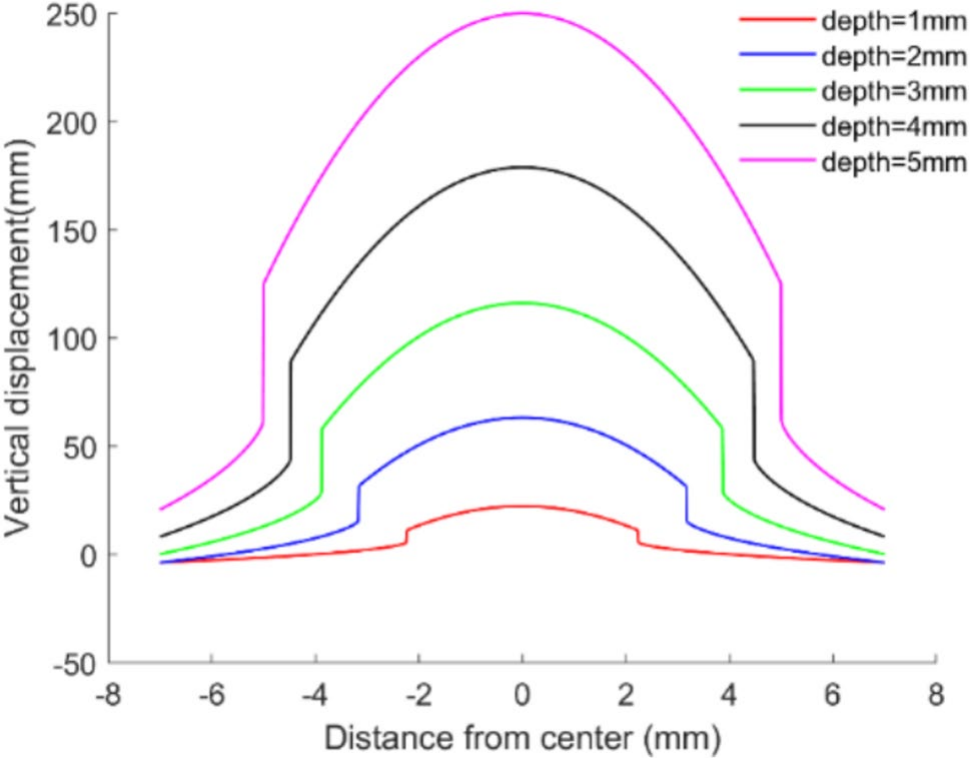
Displacement of skin dependent on the indentation depth (sphere radius is 5 mm)

Based on above, we assume the force is applied at the center of the fingertip skin. According to the contact mechanics model (formula (1)-(7)), effect of indentation depth on the displacement of skin can be calculated and depicted in Fig. 4, which suggests that the shallower the indentation depth is, the smaller the skin displacement along the normal direction is. It suggests that with change of the force acting on the sphere, skin deformation shows different. When the force is fixed, the farther away from the contact center, the smaller the skin deformation is. When the distance from the force application is fixed, the greater the pressure becomes, the deeper the deformation is. Namely, deeper indentation can cause greater strain on the fingertip skin, while strain is closely related to the spiking and energy of neuron.

### A comparison between W-Z model and HH model

**Fig. 5:**
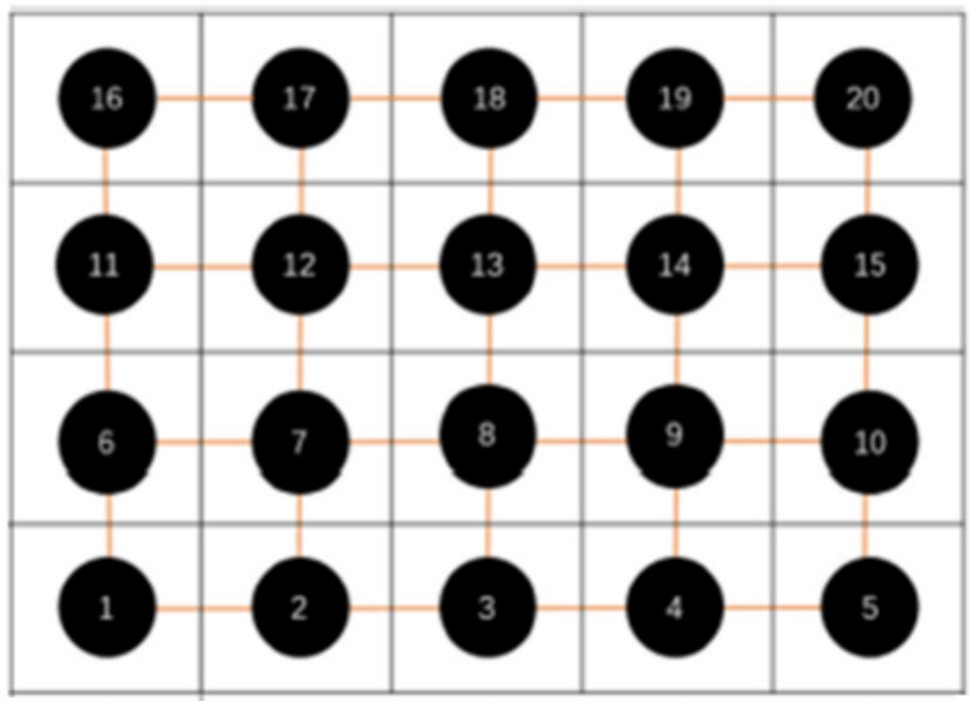
Schematic diagram of a structural network of the MCNC of glabrous skin(Yao and Wang 2019)

**Fig. 6:**
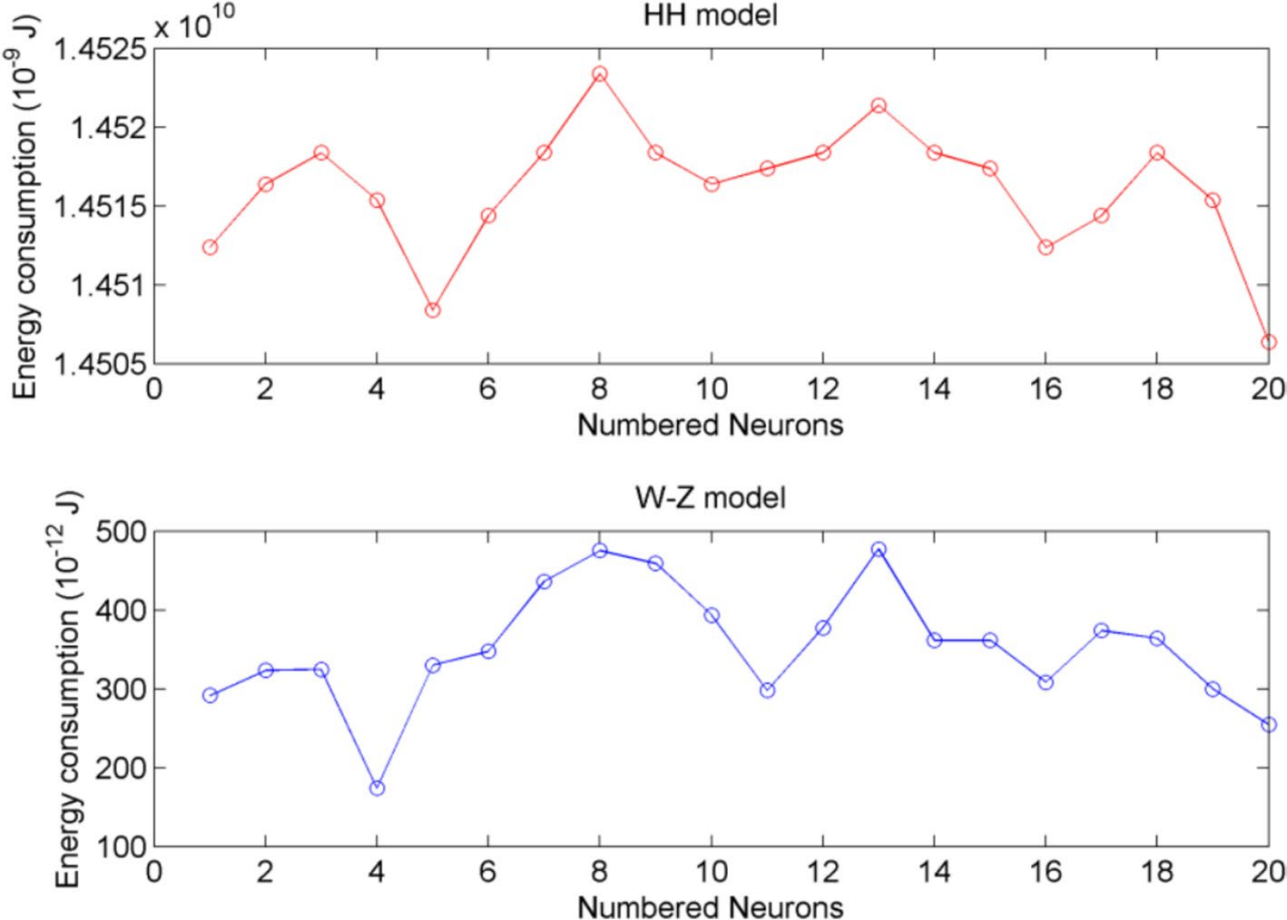
Energy consumption of neurons in networks (Yao and Wang 2019) comprising HH and W-Z models, respectively. The upper is with HH model as nodes, which is derived from integrating the power of ion channel currents (sodium, potassium, leak currents). The lower is with W-Z model as nodes using membrane potential, inductance, and resistance-based formulas

In reference (Yao and Wang 2019), the authors constructed a network consisting of 20 HH neurons by dividing the fingertip into 20 equal-area subregions (Fig. 5). It was supposed that the connection strength between nodes is randomly distributed in the range of [0.1, 0.3] and the force application point is located at the center of the area. When a sphere with 5 mm radius is squeezed into the skin of the fingertip with an extrusion of depth 3 mm, considering the schematic diagram in Fig. 5, the nodes are taken as HH model and W-Z model, respectively, and the energy consumption of the neurons can be calculated (shown in Fig. 6). Figure 6 indicates that, compared to the HH model, W-Z model requires less energy consumption. In Fig. 6, it can also be found that energy fluctuations stem from neuron position. The neurons near the contact center (e.g., Nos. 8, 13) experience stronger compression, leading to more active firing and higher energy consumption, while peripheral neurons have weaker stimulation and lower consumption, resulting in position-dependent fluctuations.

**Fig. 7:**
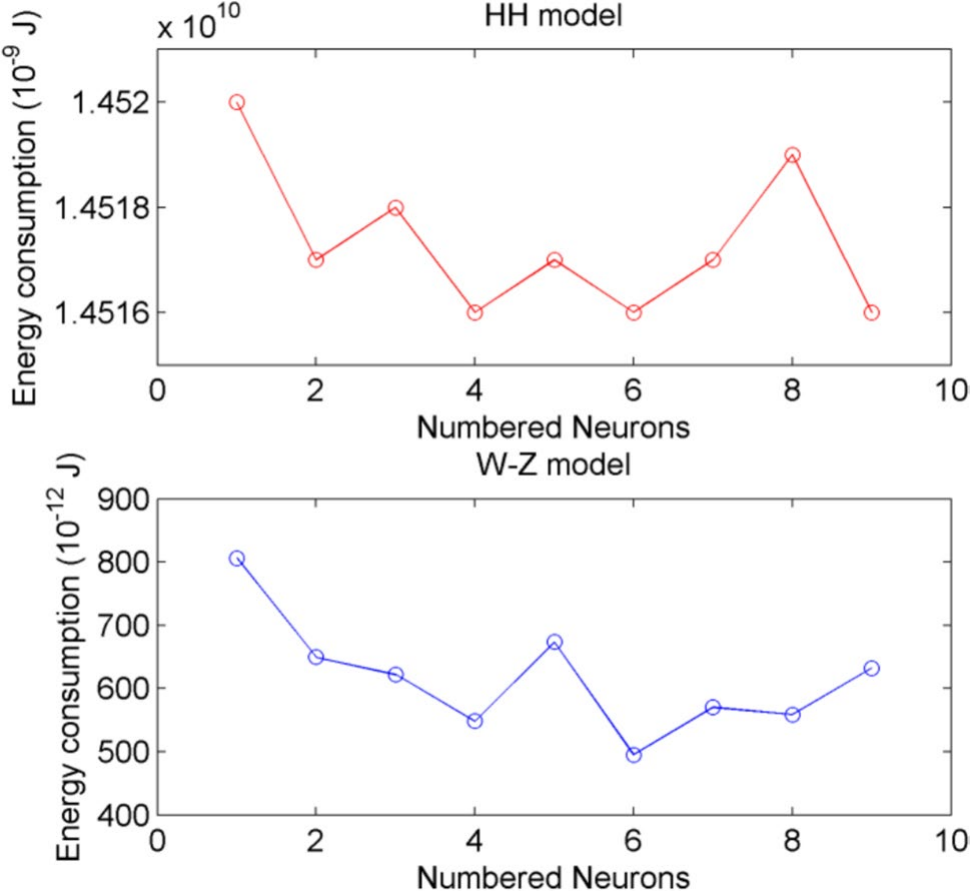
Energy consumption of neurons in star-like networks comprising HH and W-Z models, respectively. The upper is with HH model as node. The lower is with W-Z model as node

Nextly, the star-like neuron network with the structure shown in Fig. 3 is consider, where the nodes are selected as HH model and W-Z model, respectively. A sphere with 5 mm radius is used. The indentation depth is chosen as $d=4mm$. This choice is well-founded that human fingertip skin is 2–5 mm thick (Gerling and Thomas 2008; Kim et al. 2010), and 4 mm indentation depth can activate deep dermal SAI mechanoreceptors without tissue damage. It aligns with the result (Sripati et al. 2006; Yao and Wang 2019) using 3–5 mm depths for meaningful tactile interactions. The star-like consisting one hub node and eight peripheral nodes, coupling strength ${w}_{ij}$ are uniformly distributed in [0.1, 0.3]. The energy consumption is depicted in Fig. 7. Figure 7 also verifies that W-Z model requires less energy consumption than HH model, which is the same as the result of Fig. 6. Figure 6 and Fig. 7 validate W-Z model’s efficiency in tactile network energy research, and comparisons with the Yao-Wang model’s firing characteristics further confirm its physiological rationality.

In Fig. 7, it is also found that the hub node accounts for much of the energy consumption due to three factors: (1) As a signal integration center, it receives input from all peripheral nodes, involving frequent synaptic transmission and active ion transmembrane transport. (2) Directly subjected to external compression, it consumes more energy to resist deformation and maintain membrane potential stability. In addition, Johansson et al. (Johansson et al. 1982) confirmed that neurons in the fingertip’s core region exhibit significantly higher firing rates and metabolic activity than peripheral neurons, which is consistent with the hub node’s characteristics in our study.

Furthermore, the energy consumption and the average firing rate of each sensory neuron when the fingertip is under pressure is given in Table 1. Table 1 demonstrates the correlation between firing rate and energy consumption observed in the star-like neuron network. The central neuron, due to the integration of multiple input signals, experiences a significantly stronger depolarization of membrane potential compared to the surrounding neurons. Its firing rate reaches 27 Hz and energy consumption is 806.24 × 10⁻^12^ J, both of which are the highest in the network. The firing rate of the surrounding neurons remains stable at 25-26 Hz, with relatively consistent energy consumption levels, and overall lower than that of the central neurons.

**Table 1:** Energy consumption and average firing rate of each neuron

Numbered neuron	1	2	3	4	5	6	7	8	9
Energy consumption (10^–12^ J)	806.24	649.52	622.15	548.24	673.62	495.26	570.13	558.63	632.32
Average firing rate	27	26	26	25	26	25	26	26	26

After fingertip squeezing stimulation, in the star-shaped neuronal network, the central neuron, due to the integration of multiple input signals, experiences a significantly stronger depolarization of membrane potential compared to the surrounding neurons. Its firing rate reaches 27 Hz and energy consumption is 806.24 × 10⁻^12^ J, both of which are the highest in the network. The firing rate of the surrounding neurons remains stable at 25-26 Hz, with relatively consistent energy consumption levels, and overall lower than that of the central neurons.

In light of the above discussion, it is known that W-Z model is inherently designed for energy calculation, which directly aligns with our focus on the “stimulation-synchronization-energy metabolism” in tactile cognition. Therefore, in following investigation, W-Z model is chosen as the network nodes and a sphere with 5 mm radius is used.

### Synchronization of the star-like neuron network

**Fig. 8:**
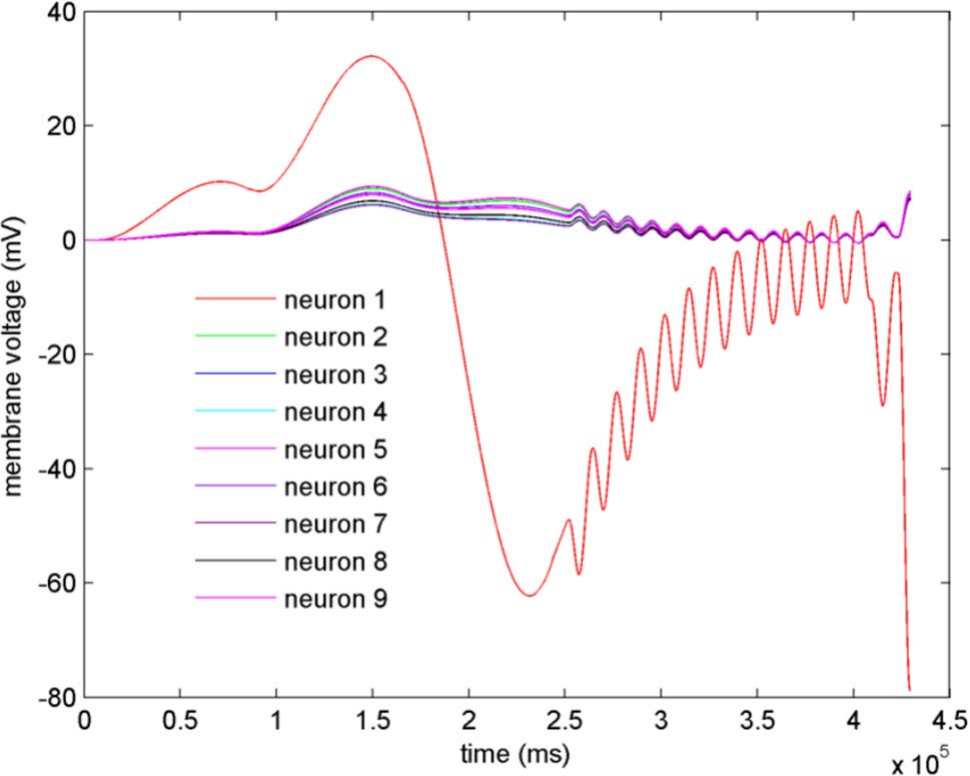
The membrane potentials of the neurons in the star-like network shown in Fig. 3 with coupling strength distributed in (0.5,0.8)

To discuss the dynamics of the star-like neuron network, two measures are mentioned, the mean-max correlation coefficient (Zhu et al. 2018) and Kuramoto order parameter (Bergner et al. 2012). Suppose the indentation depth $d=4\mathrm{mm}$, and the star-like is adopted the structure in Fig. 3. Coupling strengths are uniformly distributed in [0.01, 0.03], [0.03,0.05], [0.05, 0.08], [0.08, 0.5], [0.5, 0.8], [0.8, 1], respectively. The mean-max correlation coefficient and Kuramoto order parameters are calculated and listed in Table 2. From Table 2, the mean-max correlation coefficient is close to 1, which means that the network’s behavior approaches a state of multi-cluster synchronous coexistence. The membrane potentials of the neurons are depicted in Fig. 8, which suggests that other neurons achieve synchronization as time going except for neuron 1. It verified the result in Table 2. Furthermore, from Table 2, it’s obvious that, the greater the coupling strength is, the larger the mean-max correlation coefficient is. Namely, the synchronization between peripheral nodes can be achieved easily with coupling strength increasing.

**Table 2:** Mean-max correlation coefficient and Kuramoto order parameters

Distribution interval of coupling strength	Mean-max correlation coefficient	Kuramoto order parameters	
		*r*_*direct*_	*r*_*indirect*_
(0.01,0.03)	0.9936	0.999	0.5
(0.03,0.05)	0.9938	0.999	0.5
(0.05,0.08)	0.9941	0.999	0.5
(0.08,0.5)	0.9956	0.999	0.5
(0.5,0.8)	0.9993	1.0	0.5
(0.8,1)	1.0	1.0	0.5

To calculate the Kuramoto order parameters of the above-mentioned star-like network composed W-Z models, the phase space reconstruction theory is used. Because the W-Z neuron model discharge state has multiple rotation centers, to calculate the phase, the attractor should be projected onto a plane with only one rotation center. In our work, the $V(t) - V(t - \tau )$ plane is discussed, where delay is $\tau = 1$, and the rotation center becomes(0, 0).Therefore, the instantaneous phase of W-Z neuron can be defined as.

17 $$ \theta (t) = \arctan (V(t - \tau )/V(t)) $$

According to mutual information method (Kantz 2001), $\tau = 1$ is determined. And then, instantaneous phase angle(Li et al. 2008) on the $V(t) - V(t - \tau )$ plane is depicted as

18 $$ \varphi (t) = \left\{ {\begin{array}{*{20}c} {\begin{array}{*{20}c} {\theta (t) + 2k\pi } & {\begin{array}{*{20}c} {} & {\begin{array}{*{20}c} {for} & {} \\ \end{array} V(t) > 0,V(t - \tau ) > 0} \\ \end{array} } \\ \end{array} } \\ {\begin{array}{*{20}c} {\pi /2 + 2k\pi } & {\begin{array}{*{20}c} {} & {\begin{array}{*{20}c} {for} & {} \\ \end{array} V(t) = 0,V(t - \tau ) > 0} \\ \end{array} } \\ \end{array} } \\ {\pi + \theta (t) + 2k\pi \begin{array}{*{20}c} {} & {\begin{array}{*{20}c} {for} & {} \\ \end{array} V(t) < 0,V(t - \tau ) > 0} \\ \end{array} } \\ {\pi + \theta (t) + 2k\pi \begin{array}{*{20}c} {} & {\begin{array}{*{20}c} {for} & {} \\ \end{array} V(t) < 0,V(t - \tau ) < 0} \\ \end{array} } \\ {3\pi /2 + 2k\pi \begin{array}{*{20}c} {} & {\begin{array}{*{20}c} {for} & {} \\ \end{array} V(t) = 0,V(t - \tau ) < 0} \\ \end{array} } \\ {2\pi + \theta (t) + 2k\pi \begin{array}{*{20}c} {} & {\begin{array}{*{20}c} {for} & {} \\ \end{array} V(t) > 0,V(t - \tau ) < 0} \\ \end{array} } \\ \end{array} } \right. $$

where the value of $k$ follows the principles.

19 $$ k = \left\{ {\begin{array}{*{20}c} {k + 1\begin{array}{*{20}c} {} & \begin{gathered} V{(}t{ - }\tau {\text{) changes from negative to positive while }} \hfill \\ V{(}t{\text{) remains positive }} \hfill \\ \end{gathered} \\ \end{array} } \\ {k\begin{array}{*{20}c} {} & {others\begin{array}{*{20}c} {\begin{array}{*{20}c} {\begin{array}{*{20}c} {\begin{array}{*{20}c} {} & {} & {} \\ \end{array} } & {} & {} & {} \\ \end{array} } & {} & {} & {} \\ \end{array} } & {} & {} & {} \\ \end{array} } \\ \end{array} } \\ \end{array} } \right. $$

**Fig. 9:**
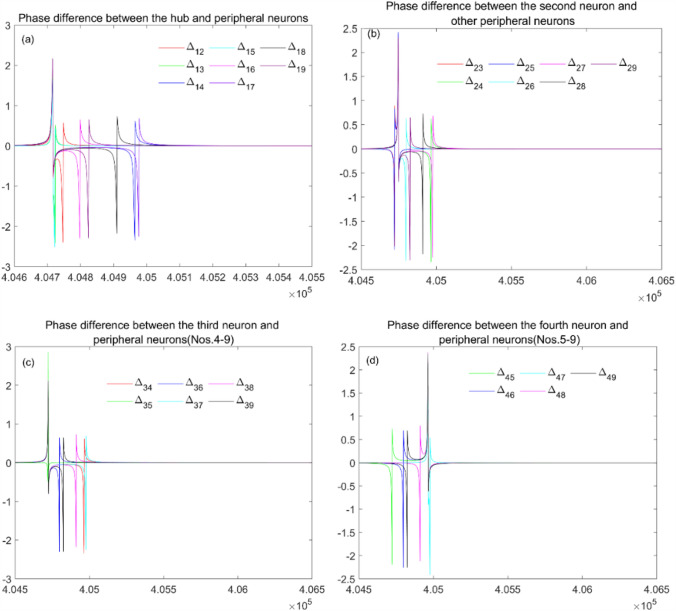
Phase difference between different neurons. In **a**, $\Delta_{1j} (j = 2,3, \cdots ,9)$ denotes the phase difference between the hub and the $j{\mathrm{th}}$ $(j = 2,3, \cdots ,9)$ neuron. In **b**, $\Delta_{2j} (j = 3,4, \cdots ,9)$ means the phase difference between the second neuron and the $j{\mathrm{th}}$ $(j = 3,4, \cdots ,9)$ neuron. In **c**, $\Delta_{3j} (j = 4,5, \cdots ,9)$ is the phase difference between the third neuron and the $j{\mathrm{th}}$ $(j = 4,5, \cdots ,9)$ neuron. In (d), $\Delta_{4j} (j = 5,6, \cdots ,9)$ means the phase difference between the fourth neuron and the $j{\mathrm{th}}$ $(j = 5,6, \cdots ,9)$ neuron

And then, phase difference between the neurons is calculated and depicted in Fig. 9, which suggests that the phase synchronization between the hub and peripheral neurons can be achieved (Fig. 9(a)). From Fig. 9 (b) (c) (d), we can see that phase synchronization has also been reached between peripheral neurons. It suggests that there are synchronous clusters in the network depicted in Fig. 3, which is consistent with the results in Table 2. By calculating the phase angle of the neurons, Kuramoto order parameters can be obtained as $r_{direct}$ and $r_{indirect}$(see Table 2). From Table 2, the remote synchronization between the hub neuron and peripheral neurons can be realized.

**Fig. 10:**
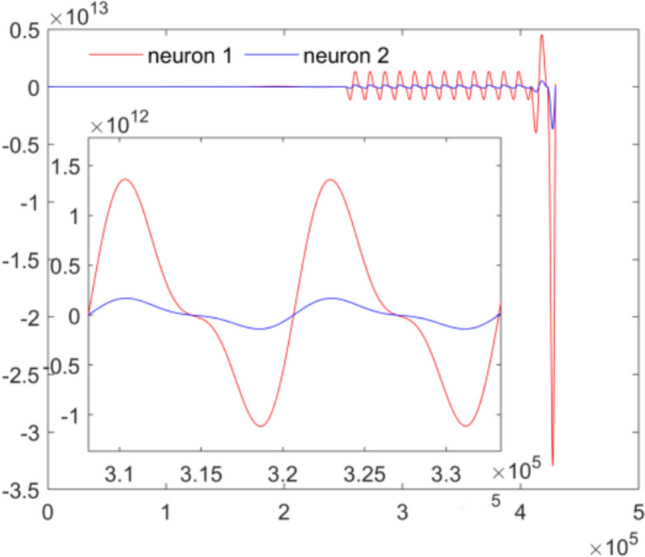
Comparison of energy consumption by the hub neuron and one peripheral neuron with unit 10^–12^ J

**Fig. 11:**
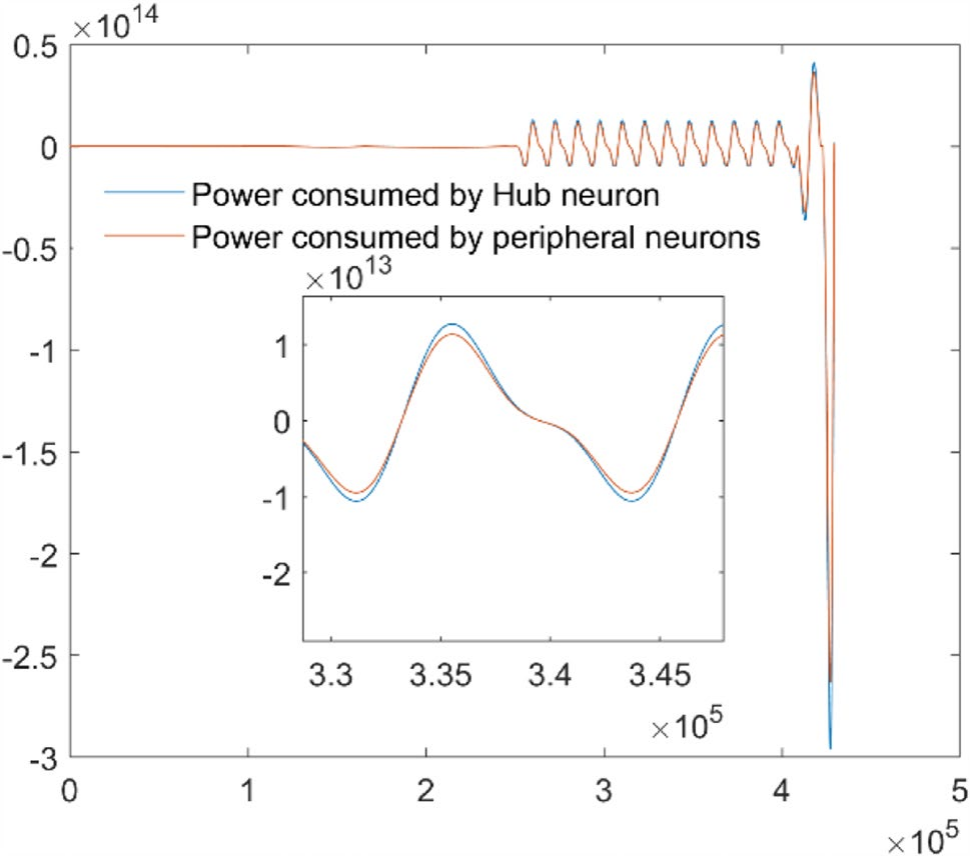
Energy consumption: hub neuron vs. peripheral neurons with unit 10^–12^ J

As we all know, during the process of neuron’s synchronization, many neurons are active simultaneously, which will induce a sharp increase in energy demand. Therefore, when the synchronization is achieved between the hub and peripheral neuron, corresponding change will occur for the energy consumed. The power consumed is given in Fig. 10 and Fig. 11. Figure 10 gives the energy consumed by neuron 1 and neuron 2, which indicates that the energy consumed by the hub neuron is much more than that of the peripheral neuron. It is mainly due to the fact that the hub neuron consumed much energy to resist the external pressure and dominate remote synchronization by adjusting its firing pattern to match peripheral nodes, increasing metabolic costs. The energy consumed by the peripheral neuron to synchronize the hub neuron is much less than that of the hub neuron. Furthermore, the total energy consumed by all peripheral neurons is calculated and depicted in Fig. 11, which indicates that the energy consumed by all peripheral neurons is also less than the energy consumed by hub neuron. Figure 10 and Fig. 11 demonstrate the pivotal role of the hub neuron in the star-like neuron network of the fingertip skin when compressed by external force. More simulation suggests that the same result can be obtained for indentation depth $d = 1{\mathrm{mm}}$,$d = 2{\mathrm{mm}}$,$d = 3{\mathrm{mm}}$ and $d = 5{\mathrm{mm}}$.

### Relationship between pressure and energy consumption of the network

**Fig. 12:**
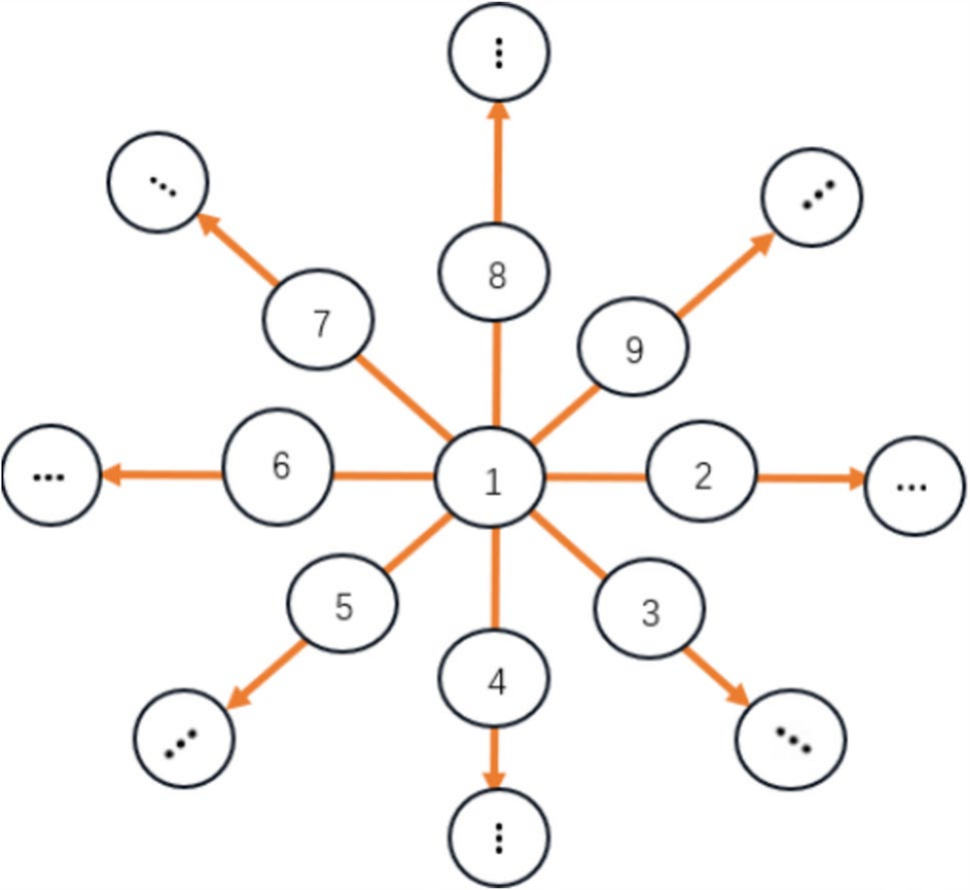
The topological structure of a multi-layer star-like neuron network

**Fig. 13:**
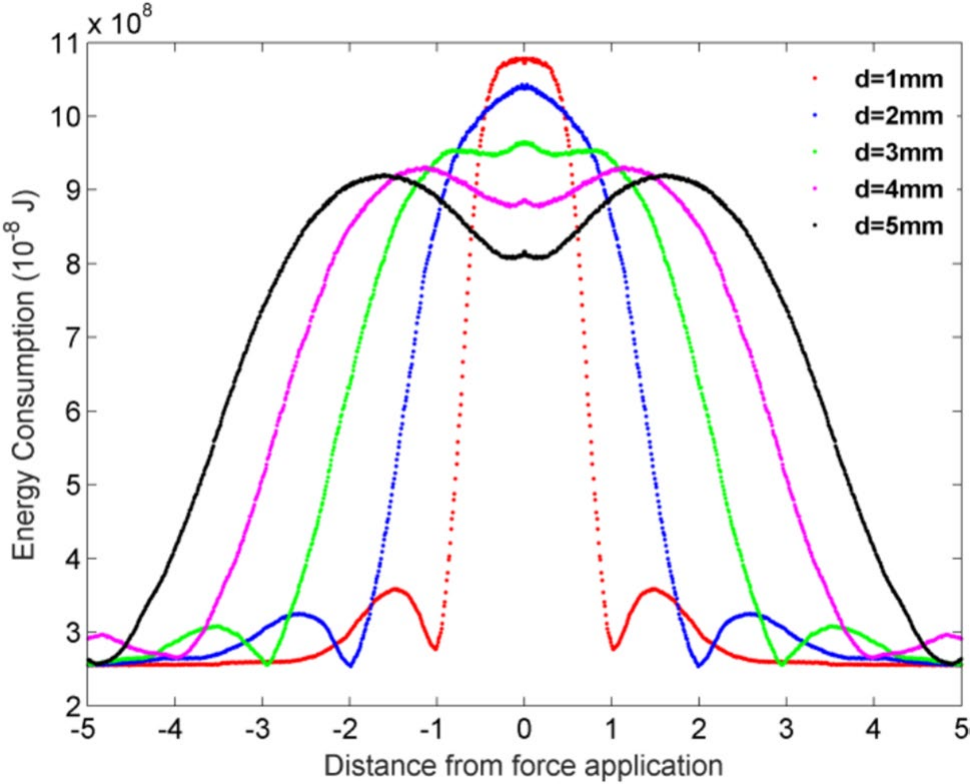
The energy consumption (10⁻⁸ J) of the neurons with a different distance from the force application

In this section, we analyze the change of energy consumption in a multi-layer star-like neural network with 8 neurons per layer(see Fig. 12). First, we also assume synaptic coupling strengths between neurons are uniformly distributed in [0.1, 0.3]. Neuron energy consumption is calculated and plotted in Fig. 13.

From Fig. 13, following results are obtained. Neuron need to consume energy to counter external pressure when the fingertip skin is compressed by a sphere. The neuron energy consumption is spatially distributed with a “high center, low on both sides” pattern centered around the point of force application, and the indentation depth (pressure) significantly regulates the peak value, distribution range, and secondary fluctuations of energy consumption. Under low pressure (*d* = 1 mm), the peak value of energy consumption is low and the distribution is the narrowest, accompanied by secondary energy peaks due to skin elastic rebound, reflecting energy conservation under weak stimulation but accompanied by additional energy expenditure. Under moderate pressure (*d* = 3 mm), the peak value of energy consumption reaches its highest level, with a moderate distribution and no secondary fluctuations, representing the optimal state of energy utilization efficiency in the tactile system, enabling precise pressure localization and signal encoding. Under high pressure (*d* = 4 ~ 5 mm), the peak value of energy consumption decreases, the distribution range significantly widens, and secondary fluctuations disappear. This spatial dispersion of energy consumption avoids excessive activation and damage to local neurons, but it is accompanied by a slight decrease in tactile localization accuracy. These results reveal the dynamic trade-off strategy between energy consumption and perceptual function in the fingertip tactile system, providing experimental evidence for the neuron energy encoding mechanism of skin pressure signals.

### The relation between the layer of the star-like network and the energy consumption

**Fig. 14:**
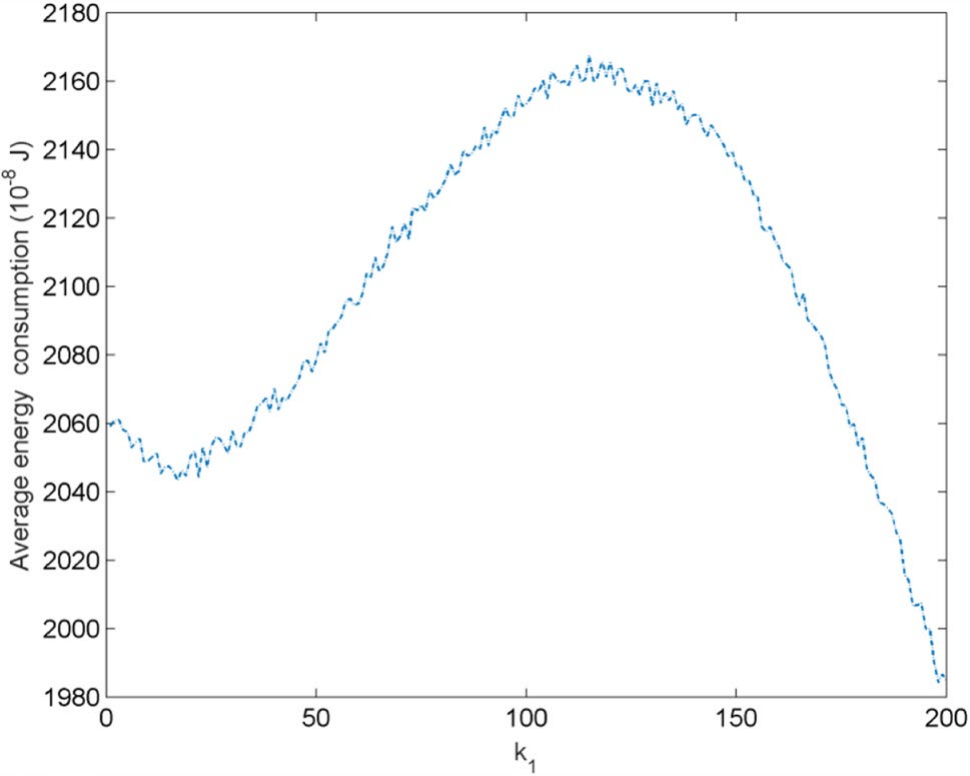
Relation between the layer and the energy consumption of the star-like network for indentation depth $d = 4{\mathrm{mm}}$, where $k_{1}$ is the number of layers of the star-like network

When the skin of the fingertip is subjected to external compression, the neuron population in the contacting center can receive stimulus. Consider the indentation depth $d = 4{\mathrm{mm}}$, the average energy consumption of the star-like neuron network with different layers can be calculated and illustrated in Fig. 14. Figure 14 illustrates how the average energy consumption of the neuron network changes with its layer count ${k}_{1}$. It is obvious that the energy consumption follows a single-peak trend: it rises as layers increase from 0 to 100, peaking at ${k}_{1}$ ≈ 100 when the network achieves the optimal balance between energy use and computational efficiency. Beyond 100 layers, energy consumption decreases due to redundancy or gradient vanishing, where deeper layers fail to contribute effectively. Namely, when the neuron network is within the contact radius, the neurons consum much energy to counter the external compression and transmit nerve impulses to peripheral neurons. As the network size increases, the neuron of the outer layer tends resting state, the energy of the network gradually decreases. It reveals a dynamic trade-off between energy expenditure and functional performance, highlighting an optimal layer count for both biological and artificial neural network design.

### The relation between the indentation depth and the energy consumption of a star -like network

**Fig. 15:**
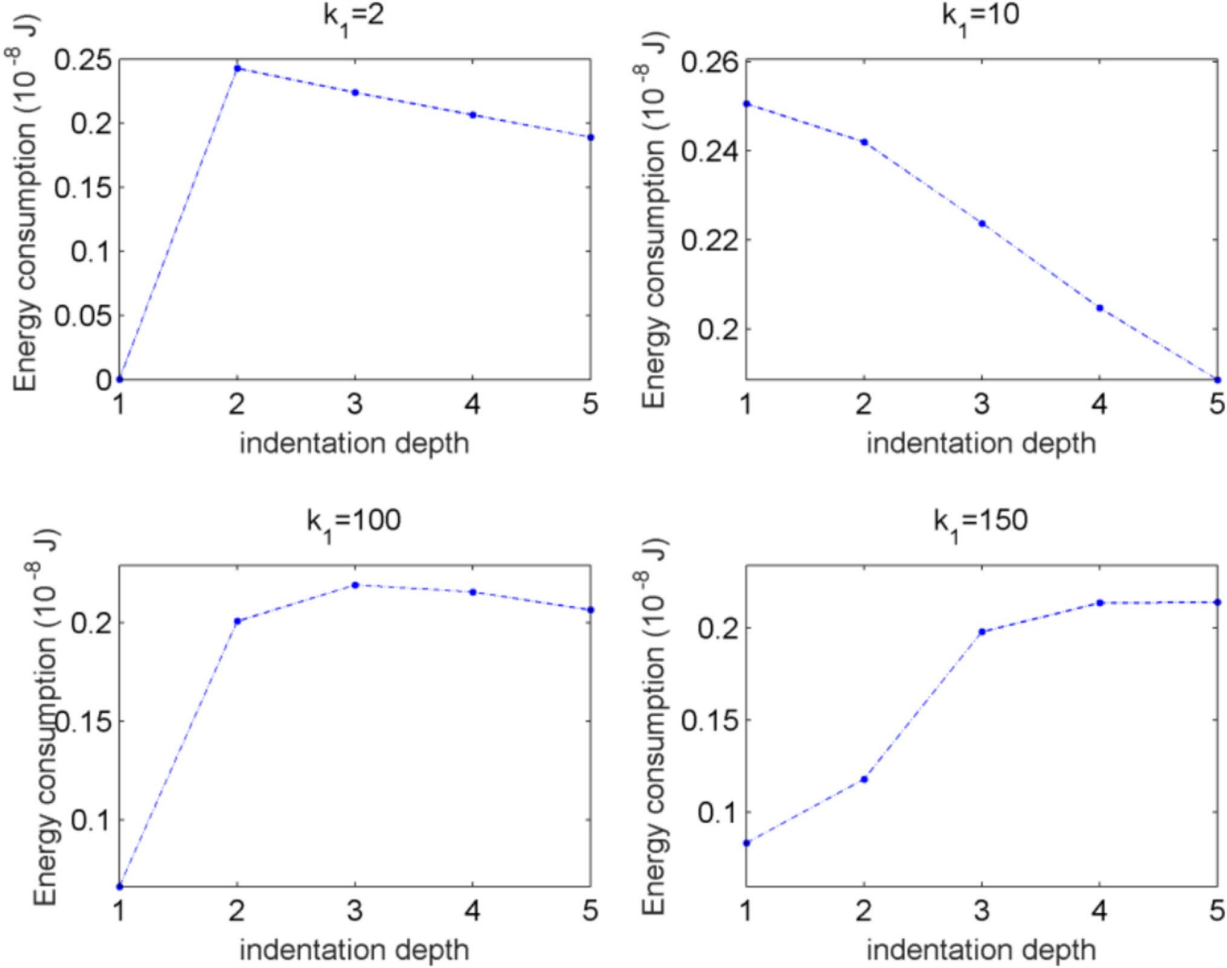
The total energy consumption of networks under different indentation depths

Consider the star-like networks with 2, 10, 100, 150 layers, simulation results for indentation depths from 1 to 5 mm are shown in Fig. 15. From Fig. 15, it can be known that networks with different layers exhibit distinct energy response patterns to pressure, and there exists an optimal number of layers (*k*₁ = 100) that strikes a balance between energy consumption and pressure encoding efficiency. For this network, energy consumption initially increases with the depth of indentation, reaches a peak at a moderate pressure (2 ~ 3), and then slowly decreases.This is consistent with the previous unimodal pattern, meaning that the network with this number of layers has the highest energy utilization efficiency under moderate pressure and can accurately encode pressure signals. When the indentation depth is small (< 2), energy consumption rapidly climbs to a peak and then slowly decreases. It means that the small network is very sensitive to initial pressure changes, but when the pressure continues to increase, energy consumption is difficult to maintain at a high level due to the limited network capacity. For a deep network(*k*₁ = 150), energy consumption continues to rise with indentation depth increasing and tends to stabilize under high pressure. This is due to deep network requires more energy to process complex pressure signals, and there is no energy decay under high pressure, indicating that its redundant structure can maintain continuous activation.

## Results

In this work, when a fingertip is compressed by a sphere, via constructing a star-like neuron network, the dynamics and energy coding of the neurons in the network are discussed. Main conclusions are depicted as follows.

### Synchronization characteristic

The central neuron and peripheral neurons in the star-like network can achieve remote synchronization. The stronger the coupling strength, the easier it is for peripheral neurons to synchronize with each other, and the overall network exhibits a state of multi-cluster synchronous coexistence.

### Energy consumption distribution

The energy consumption of the central neuron is much higher than that of the peripheral neurons. This is because central neuron not only directly bear external pressure, but also serve as signal integration centers regulating synchronization processes. The overall energy consumption of neurons exhibits a spatial distribution pattern of “high in the center and low in the periphery”.

### The correlation between pressure and energy

The depth of pressure applied to the fingertip significantly affects the energy consumption of neurons. Under moderate pressure, the peak energy consumption is the highest, indicating optimal energy utilization efficiency in the tactile system, which enables precise pressure localization and signal encoding. Under low or high pressure conditions, the peak energy consumption decreases, and the distribution range of energy consumption expands under high pressure.

### Optimal value of network layers

The star-like neuron network has an optimal number of layers (approximately 100 layers). At this number of layers, the energy consumption and information processing efficiency of the network achieve the best balance; too many or too few layers will lead to decreasing in energy utilization efficiency.

## Data Availability

No datasets were generated or analysed during the current study.

## References

[CR1] Agustin FM, Pablo PL, Gerardo J et al (2024) Neuron configuration enhances the synchronization dynamics in ring networks with heterogeneous firing patterns. Chaos Soliton Fract 187:115461. 10.1016/j.chaos.2024.115461

[CR2] An XL, Jiang LF, Xiong L et al (2024) Synchronization behavior and energy evolution in physical neuron and network. Nonlinear Dyn 112(18):16389–16407

[CR3] Batista CAS, Viana RL, Lopes SR et al (2014) Dynamic range in small-world networks of Hodgkin-Huxley neurons with chemical synapses. Phys A 410:628–640. 10.1016/j.physa.2014.05.069

[CR4] Bergner A, Frasca M, Sciuto G et al (2012) Remote synchronization in star networks. Phys Rev E 85(2):026208. 10.1103/PhysRevE.85.026208 10.1103/PhysRevE.85.026208 22463300

[CR5] Brittin CA, Cook SJ, Hall DH et al (2021) A multi-scale brain map derived from whole-brain volumetric reconstructions. Nature 591:105–110. 10.1038/s41586-021-03284-x 33627874 10.1038/s41586-021-03284-x PMC11648602

[CR6] Deng B, Wang L, Wei XL et al (2014) Network effect on the enhancement of stochastic resonance in a randomly connected neural network. 11th World Congress on Intelligent Control and Automation (WCICA) 5457–5462.

[CR7] Ding DW, Wang MY, Wang J et al (2024) Dynamic behaviors analysis of fraction-order neural network under memristive electromagnetic induction. Acta Phys Sin 73(10):100502. 10.7498/aps.73.20231792

[CR8] Elliott T (2022) The impact of sparse coding on memory lifetimes in simple and complex models of synaptic plasticity. Biol Cybern 116(3):327–362 35286444 10.1007/s00422-022-00923-y PMC9170679

[CR9] Gal E, Amsalem O, Schindel A et al (2021) The role of hub neurons in modulating cortical dynamics. Front Neural Circuits 15:718270. 10.3389/fncir.2021.718270 34630046 10.3389/fncir.2021.718270 PMC8500625

[CR10] Gerling GJ, Thomas GW (2008) Fingerprint lines may not directly affect SAI mechanoreceptor response. Somatosens Mot Res 25(1):61–76. 10.1080/08990220701838996 18344148 10.1080/08990220701838996

[CR11] Iggo A, Muir AR (1969) The structure and function of a slowly adapting touch corpuscle in hairy skin. J Physiol 200(3):763–796. 10.1113/jphysiol.1969.sp008721 4974746 10.1113/jphysiol.1969.sp008721 PMC1350526

[CR12] Intveld RW, Dann B, Michaels JA et al (2018) Neural coding of intended and executed grasp force in macaque areas AIP, F5, and M1. Sci Rep 8:17985 30573765 10.1038/s41598-018-35488-z PMC6301980

[CR13] Jo SH, Yin HJ, Mao ZH (2005) Random neural networks with state-dependent firing neurons. IEEE Trans Neural Netw 16(4):980. 10.1109/TNN.2005.849829 16121738 10.1109/TNN.2005.849829

[CR14] Johansson RS, Landstro¨m U, Lundstro¨m R (1982) Sensitivity to edges of mechanoreceptive afferent units innervating the glabrous skin of the human hand. Brain Res 244(1):27–35. 10.1016/0006-8993(82)90900-3 6288181 10.1016/0006-8993(82)90900-3

[CR15] Johnson KO (2001) The roles and functions of cutaneous mechanoreceptors. Curr Opin Neurobiol 11:455–461. 10.1016/S0959-4388(00)00234-8 11502392 10.1016/s0959-4388(00)00234-8

[CR16] Joseph D, Kumar R, Karthikeyan A et al (2024) Dynamics, synchronization and traveling wave patterns of flux coupled network of Chay neurons. Biosystems 235:105113. 10.1016/j.biosystems.2023.105113 38159671 10.1016/j.biosystems.2023.105113

[CR17] Kantz H (2001) Time series analysis in reconstructed state space. Stoch Dyn 1:85–111

[CR18] Kim EK, Gerling GJ, Wellnitz SA et al (2010) Using force sensors and neural models to encode tactile stimuli as spike-based responses. Proceed Symp Haptic Interf Virt Environ Teleoper Syst. 10.1109/HAPTIC 10.1109/HAPTIC.2010.5444657 PMC3151443 21826287

[CR19] Levy WB, Baxter RA (1996) Energy efficient neural codes. Neural Comput 8(3):531–543. 10.1162/neco.1996.8.3.531 8868566 10.1162/neco.1996.8.3.531

[CR20] Levy WB, Baxter RA (2002) Energy-efficient neuronal computation via quantal synaptic failures. J Neurosci 22(11):4746 12040082 10.1523/JNEUROSCI.22-11-04746.2002 PMC6758790

[CR21] Li HQ, Xue W, Wang CQ et al (2008) Phase synchronization investigation of the electrically coupled neurons. Acta Bioch Biophys Sin 24(1):29–36

[CR22] Li F, Li D, Wang C et al (2024) An artificial visual neuron with multiplexed rate and time-to-first-spike coding. Nat Commun 15(1):3689. 10.1038/s41467-024-48103-9 38693165 10.1038/s41467-024-48103-9 PMC11063071

[CR23] Liu JJ, Liu C, Zheng ZG (2024) Heterogeneity-induced competitive firing dynamics in balanced excitatory-inhibitory spiking neuron networks. Chaos Solitons Fractals 186:115282. 10.1016/j.chaos.2024.115282

[CR24] Ma J (2023) Biophysical neurons, energy, and synapse controllability: a review. J Zhejiang Univ Sci A 24(2):109–129. 10.1631/jzus.A2200469

[CR25] Moujahid A, d’Anjou A, Torrealdea F et al (2011) Energy and information in Hodgkin-Huxley neurons. Phys Rev E 83(3):031912. 10.1103/PhysRevE.83.031912 10.1103/PhysRevE.83.031912 21517530

[CR26] Nicholas AL, Garrett BS (2004) Encoding of natural scene movies by tonic and burst spikes in the lateral geniculate nucleus. J Neurosci 47:10731–10740 10.1523/JNEUROSCI.3059-04.2004 PMC6730113 15564591

[CR27] Peters JF, Tozzi A, Ramanna S et al (2017) The human brain from above: an increase in complexity from environmental stimuli to abstractions. Cogn Neurodyn 11(1):1–4. 10.1007/s11571-017-9428-2 28761558 10.1007/s11571-017-9428-2 PMC5509610

[CR28] Phillips JR, Johnson KO (1981) Tactile spatial resolution. II.Neural representation of bars, edges, and gratings in monkey primary afferents. J Neurophysiol 46(6):1192–1203. 10.1152/jn.1981.46.6.1192 6275041 10.1152/jn.1981.46.6.1192

[CR29] Qin HX, Ma J, Wang CN et al (2014) Autapse-induced target wave, spiral wave in regular network of neurons. Sci China Phys Mech Astron 57(10):1918–1926. 10.1007/s11433-014-5466-5

[CR30] Qu JY, Wang RB (2017) Collective behavior of large-scale neural networks with GPU acceleration. Cogn Neurodyn 11:553–563. 10.1007/s11571-017-9446-0 29147147 10.1007/s11571-017-9446-0 PMC5670084

[CR31] Rubinov M, Sporns O, Thivierge JP et al (2011) Neurobiologically realistic determinants of self-organized criticality in networks of spiking neurons. PLoS Comput Biol 7(6):e1002038. 10.1371/journal.pcbi.1002038 21673863 10.1371/journal.pcbi.1002038 PMC3107249

[CR32] Shunichi A, Hiroyuki N (2005) Difficulty of singularity in population coding. Neural Comput 17(4):839–858. 10.1162/0899766053429426 15829091 10.1162/0899766053429426

[CR33] Sripati AP, Bensmaia SJ, Johnson KO (2006) A continuum mechanical model of mechanoreceptive afferent responses to indented spatial patterns. J Neurophysiol 95(6):3852–3864. 10.1152/jn.01240.2005 16481453 10.1152/jn.01240.2005 PMC1839063

[CR34] Towlson EK, Ve´ rtes PE, Ahnert SE et al (2013) The rich club of the *C. elegans* neuronal connectome. J Neurosci 33:6380–6387. 10.1523/JNEUROSCI.3784-12.2013 23575836 10.1523/JNEUROSCI.3784-12.2013 PMC4104292

[CR35] Wang ZY, Wang RB (2014) Energy distribution property and energy coding of a structural neural network. Front Comput Neurosci 8:14. 10.3389/fncom.2014.00014 24600382 10.3389/fncom.2014.00014 PMC3930871

[CR36] Wang RB, Zhang ZK, Chen GR (2009) Energy coding and energy functions for local activities of the brain. Neurocomputing 73(1–3):139–150. 10.1016/j.neucom.2009.02.022

[CR37] Wang RB, Wang ZY, Zhu ZY (2018) The essence of neuronal activity from the consistency of two different neuron models. Nonlinear Dyn 92:973–982. 10.1007/s11071-018-4103-7

[CR38] Wong W (2020) On the rate coding response of peripheral sensory neurons. Biol Cybern 114(6):609–619. 10.1007/s00422-020-00848-4 33289878 10.1007/s00422-020-00848-4

[CR39] Xu Q, Liu T, Ding SK et al (2023) Extreme multistability and phase synchronization in a heterogeneous bi-neuron Rulkov network with memristive electromagnetic induction. Cogn Neurodyn 17(3):755–766. 10.1007/s11571-022-09866-3 37265650 10.1007/s11571-022-09866-3 PMC10229522

[CR40] Yang F, Wang Y, Ma J (2023) An adaptive synchronization approach in a network composed of four neurons with energy diversity. Indian J Phys 97(7):2125–2137

[CR41] Yao MQ, Wang RB (2019) Neurodynamic analysis of Merkel cell-neurite complex transduction mechanism during tactile sensing. Cogn Neurodyn 13:293–302. 10.1007/s11571-018-9507-z 31168333 10.1007/s11571-018-9507-z PMC6520415

[CR42] Yao Z, Sun KH, Wang HH (2024) Collective behaviors of fractional-order FithzHugh-Nagumo network. Physica A 639:129673

[CR43] Yuan ZY, Wu YL, Ou CY et al (2025) Dynamical behavior of SW-SW neural networks. Chin J Phys 94:108–120. 10.1016/j.cjph.2024.12.031

[CR44] Zhang GX, Rong HN, Neri F et al (2014) An optimization spiking neural P system for approximately solving combinatorial optimization problems. Int J Neural Syst 24(5):1440006. 10.1142/S0129065714400061 24875789 10.1142/S0129065714400061

[CR45] Zhu ZY, Wang RB, Zhu FY (2018) The energy coding of a structural neural network based on the Hodgkin-Huxley model. Front Neurosci 12:122 29545741 10.3389/fnins.2018.00122 PMC5838014

